# Defectronics based photoelectrochemical properties of Cu^2+^ ion doped hematite thin film

**DOI:** 10.1038/s41598-022-20045-6

**Published:** 2022-12-05

**Authors:** Chang Woo Kim, Amol U. Pawar, Thomi Hawari, Na Hyeon Ahn, Don Keun Lee, Long Yang, Ramesh Poonchi Sivasankaran, Jun Tang, Zhongbiao Zhuo, Young Soo Kang

**Affiliations:** 1 Department of Nanotechnology Engineering, College of Engineering, Pukyong National University, Busan, 48513 Republic of Korea; 2 Environmental and Climate Technology, Korea Institute of Energy Technology (KENTECH), Naju-si, Jeollanam-do 58219 Republic of Korea; 3 Department of Chemistry, Sogang University, Seoul, 04107 Republic of Korea; 4 Zhejiang Coloray Technology Development Co., Ltd., No. 151, Huishan Road, Deqing County, Huzhou, 313200 China

**Keywords:** Electrocatalysis, Photocatalysis

## Abstract

The concentration of guest elements (dopants) into host materials play an important role in changing their intrinsic electrical and optical properties. The existence of hetero-element induce defect in crystal structure, affecting conductivity. In the current work, we report Cu^2+^ ion into hematite in the defectronics point of view and their photoelectrochemical properties. Crystal distortion in the structure of hematite is observed as the amount of dopant increases. Among 1, 3 and 5 mol% of Cu^2+^ doped hematite, the existence of 1 mol% of Cu^2+^ ion into hematite crystal structure produce photocurrent value of 0.15 mA/cm^2^, IPCE value of ~ 4.7% and EIS value of ~ 2000 Ω/cm^2^ as best performances. However, further increasing dopants increases the number of interstitial defects, which cause the deformation of intrinsic lattice structure.

## Introduction

Hematite (α-Fe_2_O_3_) has been investigated intensively as a candidate of photoelectrochemical (PEC) electrodes for water oxidation due to its favourable optical band gap of 2.0 eV allowing the high absorption of visible light with appropriate band gap, electrochemically stable, low toxic and low cost^1–4^. The PEC performance was restricted by the fact that they have a short electron diffusion length as 2–4 nm despite the above advantages. It results in a fast recombination between photogenerated electrons and holes. Finally, a hydrogen evolution rate is sluggish considering the fact that conduction band of hematite is located below that of the H^+^/H_2_ reduction potential^5–7^.

As a strategy for enhanced performances of semiconducting photocatalyst, Introduction of hetero-element doping into metal oxide crystal structure have been widely reported^8–10^. Because this approach can modify the electronic structure to change optical and catalytic properties^11^. Till date, tremendous work has been successfully proved that kinds and concentration of dopant have an effect to the enhancement in the conductivity of hematite crystal structure by adding transition metal ions such as Sn, Si, Ti, Mg, Mo, Pt and Cr^5–7,12–20^. For example, Sn-dopant was incorporated into hematite by sintering hematite nanowires in air which was grown on FTO via hydrothermal method^13^. Sn plays a role as an electron donor and thereby increases carrier density of hematite, which enhances over the increase of energy barrier by inducing defects. As a result, Sn-doped hematite recorded a photocurrent density of 1.86 mA/cm^2^ at 1.23 V vs RHE, higher than pristine hematite nanowires. Si-doped hematite thin film prepared by APCVD yielded higher IPCE value of 42% at 370 nm at 1.23 V vs RHE than pristine hematite electrodes. Especially, Grätzel group reported that the higher efficiency was enhanced by the change of crystal structure in hematite^14^. Applying insulating under-layers also has been approached for enhancement for the photocurrent density. The introduction of insulating SiO_2_ under-layer into the hematite thin film has an effect to increase the photocurrent up to − 0.76 mA at 1.23 V vs RHE under 1-sun illumination^21^. This insulating layer has shown the ability to suppress the back electron transfer from the substrate, and results in decreasing the charge recombination process. Such efforts help to enhance PEC performances and it has been related to the scientific point of how to elongate photogenerated charge. Kinds and concentration of guest elements into host materials play an important role in changing their intrinsic electrical and optical properties. Hwang et al. reported Cu-doped into flower like hematite for PEC water splitting application. It was observed that 1 mol% Cu doping enhanced the p-type nature of hematite film and increase photocurrent in cathodic region. However, the other important effects arise due to Cu doping on hematite film such as the change in photo-electro response on n-type of hematite film, defectronics based dipole moment, surface polarizability and stress in crystal lattice have not been discussed in previous studies^22^.

In the present study, we report the role of the guest element into hematite photoanode and the relationship between concentration of dopant and PEC performances. Compared with previous work, current work demonstrates that the existence of hetero-element induce defect in crystal structure, affecting conductivity in the view point of defectronics and their photoelectrochemical properties. The different mol% of Cu^2+^ ion was doped into hematite nanocrystal thin film prepared by the hydrothermal method. The prepared Cu^2+^ ion doped hematite thin film was characterized on the composition, crystal structure, and the optical and PEC performances. The observed characteristics were comparatively discussed on the relationship between concentration of dopant and crystallographic change. We observed that the longer crystal axis length and crystallinity of the Cu^2+^ ion doped hematite thin film influences the diffusivity of the photo-ejected electrons in the hematite nanocrystal thin film. The decreased optical absorption coefficients of the doped hematite thin film also decrease the photocurrent density of the Cu^2+^ ion doped hematite thin film due to a decreased surface dipole moment.

## Experimental section

### Materials

Iron chloride hexa-hydrate (Extra pure, Junsei Chemical), sodium hydroxide (extra pure, Junsei Chemical), copper nitrate hemi(penta-hydrate) (98%, Sigma-Aldrich) were used without further purification. All reagents were used without any further purification. F-doped tin oxide (FTO) coated glass (20 mm × 20 mm) was obtained from Pilkington (Toledo, OH). Deionized water (DIW, > 18 MW, Millipore) was used as a solvent for the precursor solution.

### Preparation of hematite thin film

F-doped tin oxide (FTO) coated glass was cleaned with sonication in mucasol solution and washed with DIW. Sodium hydroxide solution (10 mL, 0.05 M of NaOH) was slowly added into 10 mL of FeCl_3_ solution (0.05 M) and stirred vigorously for 10 min at room temperature (RT). When the color of the solution gradually turned dark, FTO glass was directly submerged into the solution. The reactor containing the solution and FTO glass was transferred to a Teflon-lined autoclave. The autoclave was heated in the oven for hydrothermal reaction at 150 °C for 4 h. After completing the reaction and autoclave reached to the room temperature naturally, hematite thin film took out from autoclave and was washed with DIW thoroughly. The cleaned film was annealed at 550 °C for 4 h under the air. After annealing, the same process was repeated for the secondary growth of the thin film^12^.

### Preparation of Cu^2+^ ion doped hematite thin film

Typically, Cu^2+^ doped hematite was prepared with minor revision of pristine hematite film. Before putting FTO glass into a mixture of FeCl_3_ and NaOH solution, copper nitrate (1, 3 and 5 mol% for Fe^3+^ ion concentration) was added to the mixed solution. Then, the solution is stirred vigorously for 10 min and hydrothermally heated in the oven 150 °C for 4 h. After reaction, Cu^2+^ ion doped hematite thin film was collected and annealed at 550 °C for 4 h under the air. Like the experimental procedure of pristine hematite film, secondary growth was treated^12^.

### Characterization

The X-ray diffraction pattern was checked to identify the crystalline structures of the hematite thin film. X-ray diffractometer (XRD, Rigaku miniFlex-II desktop) was used with Cu-Kα radiation as X-ray source and scanned 20–80° at a speed of 1° min^−1^. The morphology and coverage of the thin film surface were characterized by field emission-scanning electron microscope (SEM, Hitachi Horiba S-4300). Cross-sectional SEM image was obtained to check thin film thickness. Raman spectra were measured by a liquid-nitrogen-cooled CCD (Symphony, Horiba Jobin Yvon)-equipped Raman spectrometer (TRIAX 550, Horiba Jobin Yvon) with 514.5 nm line emitting Ar^+^ ion laser (Stabolite 2017, Spectra-Physics). The wavenumber was calibrated by checking the Raman peak position of Si. The laser power was kept at 50 mW for 10 s. X-ray photoelectron spectroscopy (XPS, Thermo VG Scientific Multitab 2000) was used to examine the surface elemental composition and the change in chemical oxidation state of the elements in the samples The exact dopant concentrations were observed by using Rigaku ZSX Primus II X-ray fluorescence spectrometer. The calculation of each element is based on the semi-quantitative analysis (SQX) method by using the fundamental parameter (FP) model. PEC characteristics of hematite thin film electrodes (diameter = 0.7 cm, dimension = 0.38 cm^2^) were investigated by using CHI 1000 potentiostat in a three-electrode cell. Three-electrode cell consists of 1.0 M NaOH (pH 13.6) as electrolyte, Ag/AgCl in 3.0 M KCl saturated as a reference electrode, Pt foil as a counter electrode, and hematite thin film as working electrode. I–V curves of hematite photoanode were scanned between − 700 and 650 mV vs Ag/AgCl at a rate of 50 mVs^−1^ and for chopped photocurrent was done at a rate of 20 mVs^−1^ with 2 s light on and off interval. The electrochemical impedance spectroscopy (EIS) was measured at 0 V vs Ag/AgCl in the range of 200,000 kHz to 100,000 mHz under light illumination. For the Mott-Schottky plot, the EIS was measured with a sinusoidal voltage perturbation of 10 mV in amplitude between 100 kHz and 100 Hz and was scanned from − 1.0 to 0 V vs Ag/AgCl. The Mott-Schottky plot was extracted at 1 kHz. The incident photon to current efficiency (IPCE) was measured at 0.23 V vs Ag/AgCl to compare the quantum efficiencies of each sample. Calculation of band gap energy and optical absorption coefficient was done based on the spectra obtained by using UV photoelectron spectrometer (ESCALAB 250Xi, Thermo Scientific) and UV–vis absorption spectroscopy (Agilent Cary 5000 UV–Vis–NIR).

## Result and discussion

**Figure 1:**
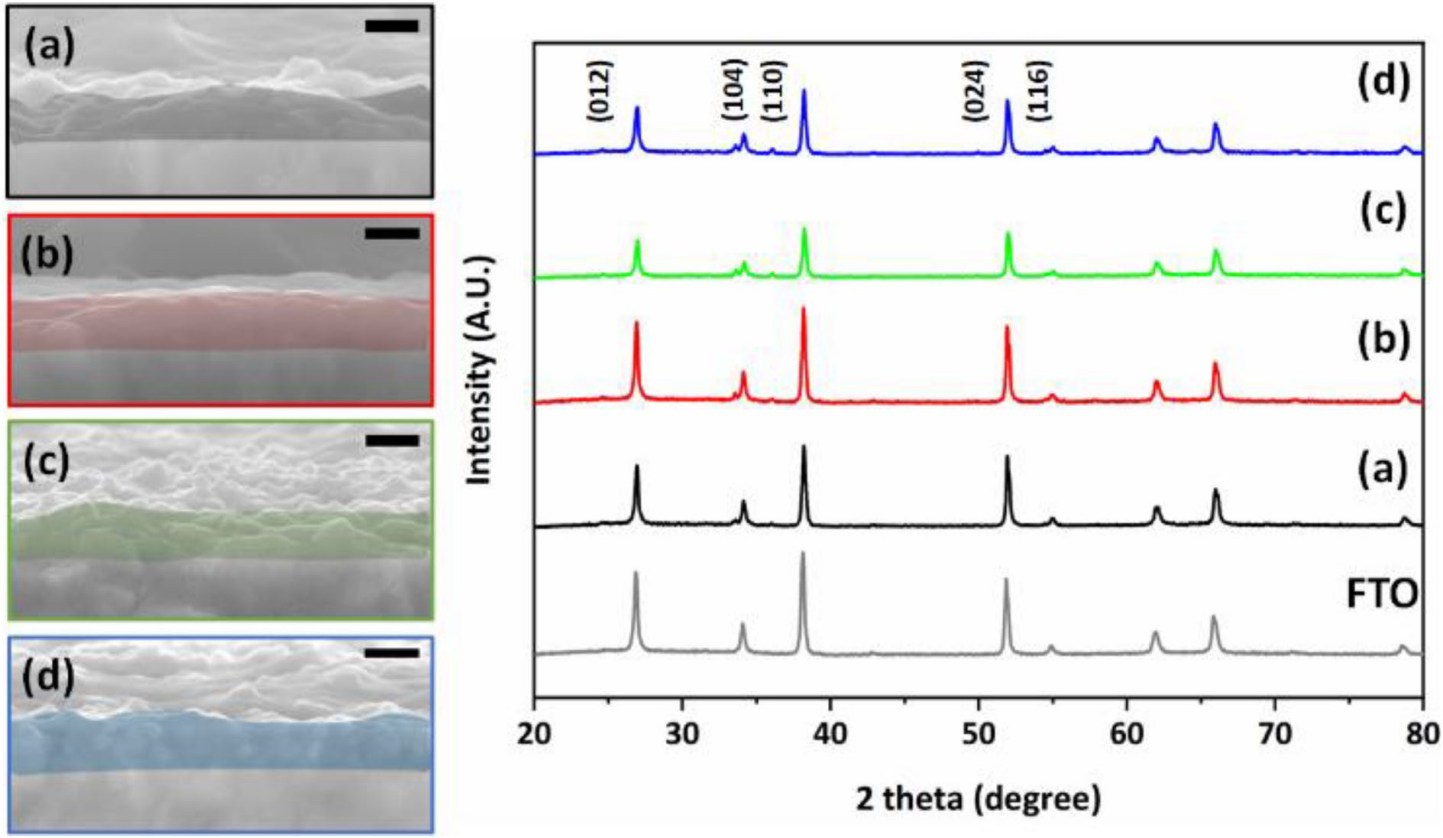
Typical SEM images and X-ray diffraction patterns of pristine hematite thin film (**a**, black) and Cu^2+^ ion doped hematite thin film with 1 mol% (**b**, red), 3 mol% (**c**, green), and 5 mol% (**d**, blue) of Cu. Black scale bar is 200 nm in SEM images.

**Figure 2:**
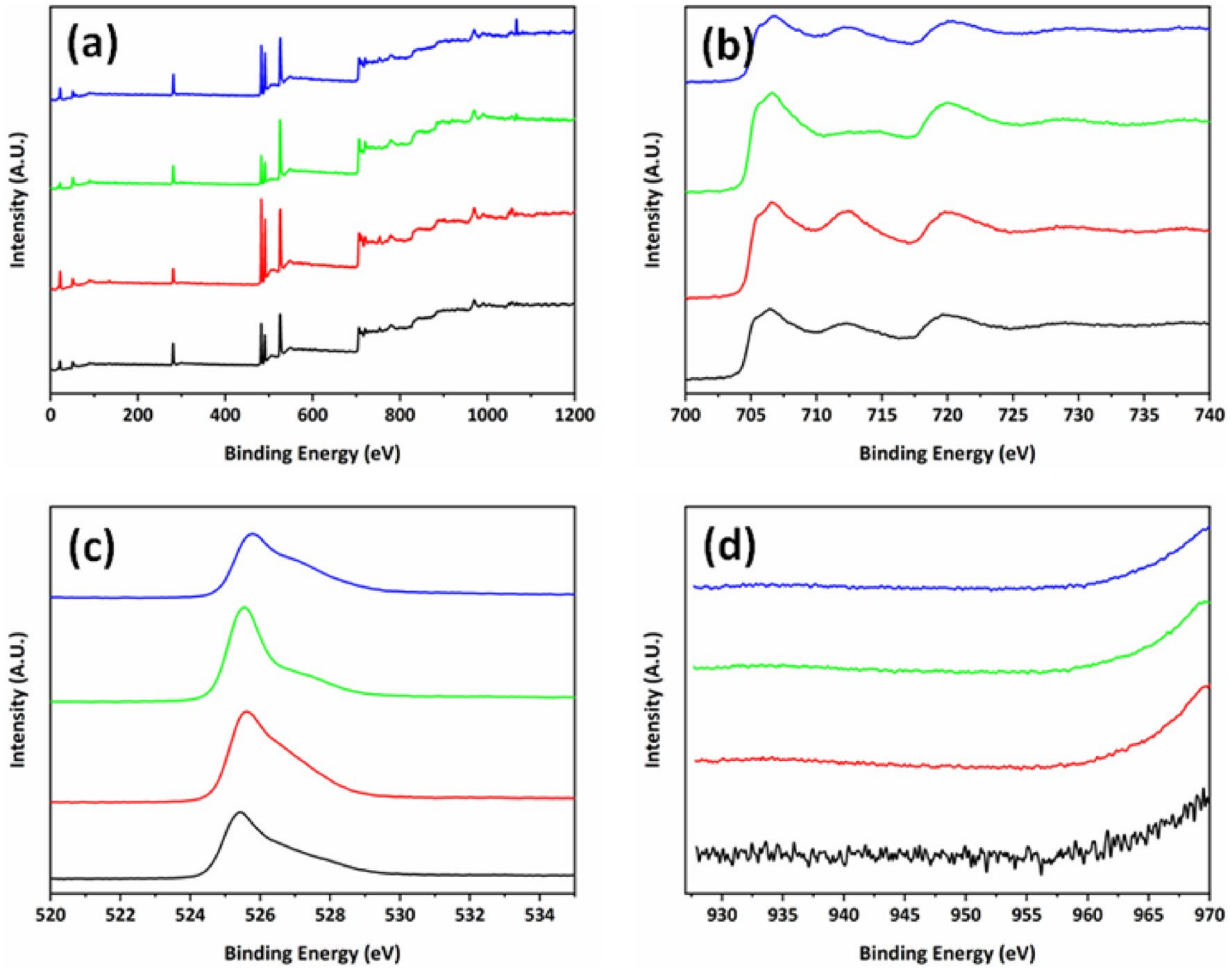
Typical XPS result of hematite thin film and Cu^2+^ ion doped hematite thin film with pristine (black), 1 mol% (red), 3 mol% (green), and 5 mol% (blue). (**a**) wide range, (**b**) Fe 2p, (**c**) O 1 s and (**d**) Cu 2p of each hematite thin films.

**Figure 3:**
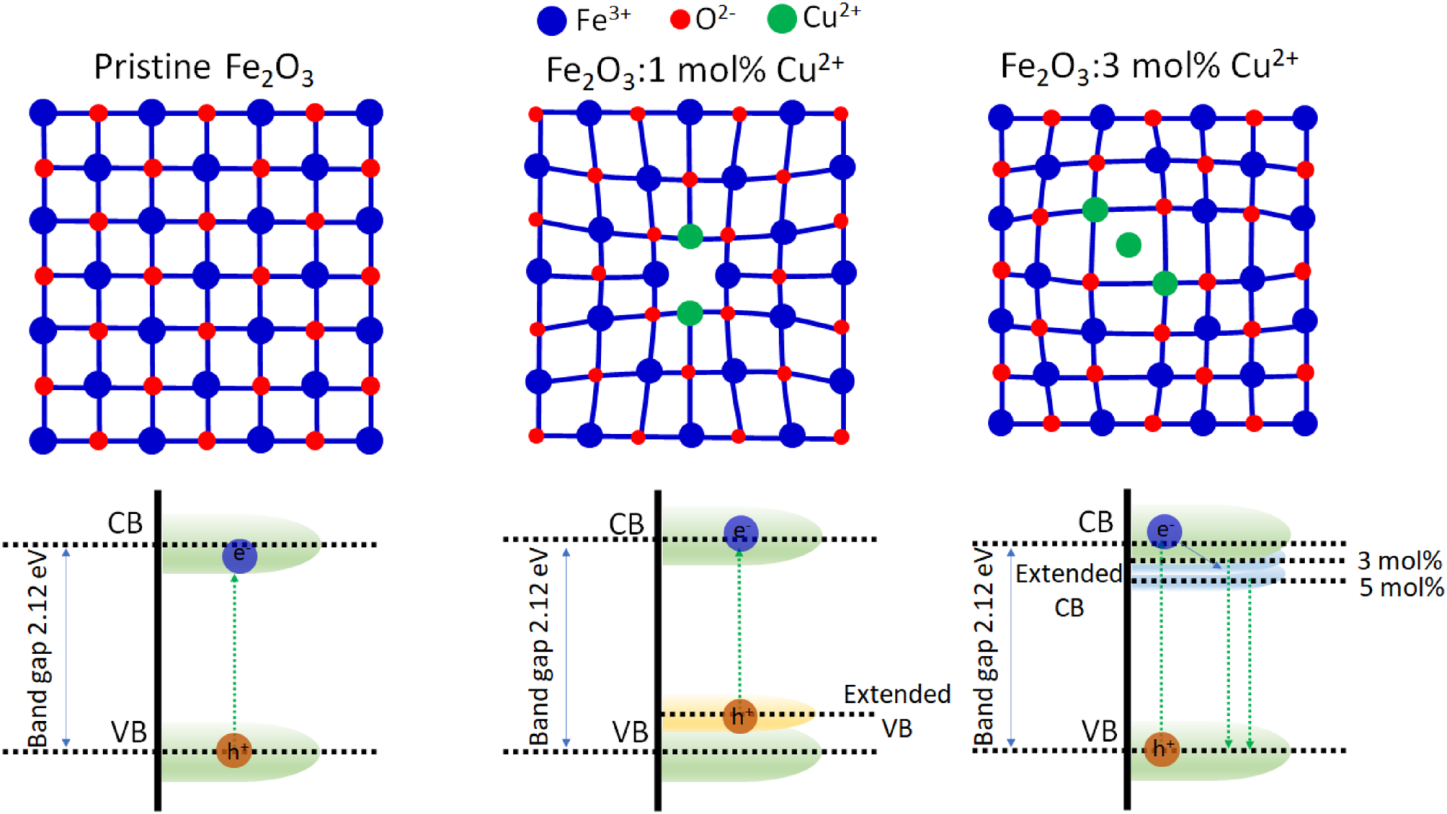
Schematic representation of defect formation and band gap modification with different amount of Cu^2+^ doping into hematite. Pristine hematite (*a*-Fe_2_O_3_) does not show any bond deformation and compression but in 1 and 3 mol% Cu^2+^ doped *a*-Fe_2_O_3_ show bond or lattice compression and deformation due to defect formation. The effect of defects on band energy diagram are presented with extended VB and CB energy level.

The crystal structure of hematite film with different kinds of Cu^2+^ ion mol% was compared with the pristine hematite thin film was investigated by cross-sectional SEM images and X-ray diffraction patterns (Fig. 1). All obtained spectra show a typical crystal structure of hematite with 2 theta values of 25°, 33°, 35°, 49.5° and 54° corresponds to planes (012), (104), (110), (024), and (116), respectively. Observed XRD peaks show very low intensity compared to FTO substrate peaks considering their crystallinity and thickness of prepared thin film samples. It shows that no significant change is observed in typical four kinds of samples considering amounts of dopants are less than 5 mol%^23–26^. The surface roughness and thickness of all the films were measured by cross sectional (Fig. 1) and top-viewed (Fig. S1) SEM images. It shows almost no change in the thickness with pristine and Cu^2+^ doped hematite thin films, the observed thickness value in coloured each image is around 200 nm (± 10 nm). It indicates that hematite thin film was uniformly covered with continuous structure on the surface of FTO substrate without any voids. The dopant concentration does not affect the shape and structure of the hematite thin films. Even though there was no critical difference in crystal structure and morphology of typical 4 kinds of hematite thin film. The crystallite size and lattice parameters of prepared hematite films are presented in Supporting Information Table S1. It was observed that crystallite size increases in 1 mol% Cu doped film (39.51 nm) and later again decreases with increasing concentration 3 mol% (34.58 nm) and 5 mol% Cu (25.14 nm) doping, however difference between them are almost negligible. X-ray fluorescence spectroscopy (XRF) analysis was performed on pristine and Cu doped Fe_2_O_3_ thin films to identify the exact amount of dopant. Obtained XRF presented in Table 1 was confirmed that Cu amount was increased to 0.32, 0.54 and 1.33 mass% with increasing dopant concentration as 1 mol%, 3 mol% and 5 mol% Cu, respectively. However, the dopant concentration is very less so it is not easily detected by some other techniques such as XPS and EDS. The oxidation state of each element in individual film was monitored by XPS. Figure 2a shows the 4 kinds of hematite thin films with pristine (black), 1 mol% Cu doped (red), 3 mol% Cu doped (green) and 5 mol% Cu doped (blue) ones. The presence of Fe 2p and O 1 s observed in small range XPS spectra and presented in Fig. 2b,c, respectively. However, the peak of Cu 2p related to Cu^2+^ in the Cu core-level spectrum (Fig. 2d) did not appear. It is presumed that current concentrations of copper dopant are under detection limit of XPS like SEM–EDS results. Given that the position of Cu^2+^ ion in the host hematite crystal structure, oxygen deficiency is calculated with the ratio between Fe and O. It is clear that the ratio between Fe and O element is related to the crystal structure distortion. Considering the α-Fe_2_O_3_ hematite with rhombohedral hexagonal crystal with R3c space group, typically dopant replaces iron positions hence two Fe^3+^ ions are replaced by two Cu^2+^ ions and the charge balance between Fe^3+^ and O^2−^ will be biased so that it induces the oxygen deficiency in the Cu^2+^ ion doped hematite thin film in Fig. 5^27^. The atomic ratios of Fe and O were found to be 0.65563 for pristine, 0.63158 for 1 mol%, 0.54464 for 3 mol% and 0.50421 for 5 mol% in the Cu^2+^ ion doped hematite thin films. It is noted that the distortion in crystal structure was found to be increased as the concentration of Cu^2+^ dopant increases. The low value of Fe/O atomic ratio indicates to induce oxygen deficiency and Cu^2+^ ion doping results in a crystal structure distortion in the Cu^2+^ ion doped hematite thin films. When the concentration of Cu^2+^ ions increases to 3 mol% and 5 mol% then extra amount of Cu^2+^ ion goes to the interstitial site of Fe_2_O_3_. It induces the partial charge balance in the crystal unit cell due to positive charge of Cu^2+^ ion and it facilitates the crystal structure distortion in Fig. 3. These crystal structure distortions make bond deformation or contraction due to defects formation with less radius of Cu^2+^ ion compared with Fe^3+^ ion. In short, in the hematite crystal structure the iron atoms are surrounded by octahedron oxygen. Hence, a replacement of Fe^3+^ ion to Cu^2+^ ion produces the oxygen distorted octahedral structure by oxygen deficiency. Finally, it results into the increase in valence band energy. Interestingly, when Cu^2+^ goes to an interstitial site, it has four oxygen atoms in tetragonal configuration instead of octahedral structure. It contributes to the lowering of conduction band energy level further with increasing Cu^2+^ concentration. Their valence and conduction bands are extended from relatively lower (1 mol%) to higher concentration (3 and 5 mol%) of doped Cu^2+^ ions by this process, which pictorial representation was given in Fig. 3.

**Table 1:** XRF elemental analysis of pristine and Cu doped Fe_2_O_3_ films.

Element	Sample (Fe_2_O_3_:Cu)			
	Pristine (Mass%)	1 mol% Cu (Mass%)	3 mol% Cu (Mass%)	5 mol% Cu (Mass%)
Cu	0	0.32	0.54	1.33
Fe	100	99.68	99.46	98.67

**Figure 4:**
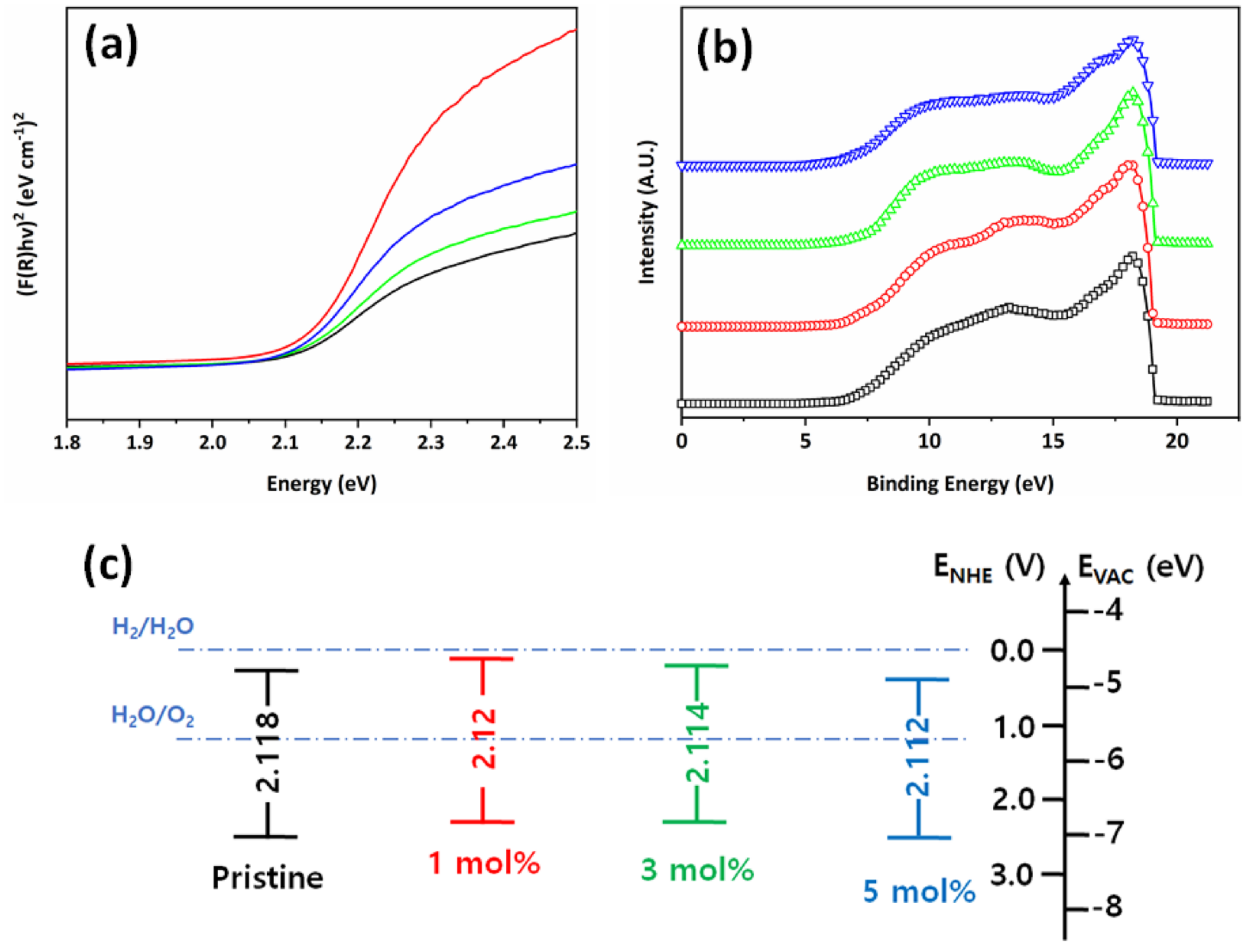
(**a**) UV–vis absorbance spectra, (**b**) UPS spectrum and (**c**) calculated bandgap diagram of Cu^2+^ ion doped hematite thin film with pristine (black), 1 mol% (red), 3 mol% (green), and 5 mol% (blue).

The experimental effect of Cu^2+^ ion dopant on optical properties in the prepared four types of hematite films were investigated by UV–visible and UPS spectroscopy. Furthermore, UV–visible absorbance data converted into Tauc plot for the band gap energy calculations in Fig. 4a and UPS data was also used to find out valence band position of pristine and Cu^2+^ ion doped hematite films in Fig. 4b. Both data were used to plot band gap diagrams with valence and conduction band positions at NHE and vacuum energy level and presented in Fig. 4c. Compared with pristine hematite with 2.118 eV, energy band gap of 1 mol% doped Cu^2+^ ion, 3 mol% doped Cu^2+^ ion and 5 mol% doped Cu^2+^ ions were calculated to 2.12 eV, 2.114 eV and 2.112 eV, respectively. Interestingly, 1 mol% of Cu^2+^ ion doped hematite thin film has a highly negative conduction band minimum and 5 mol% of Cu^2+^ ion doped film has a highly positive valence band maximum.

**Figure 5:**
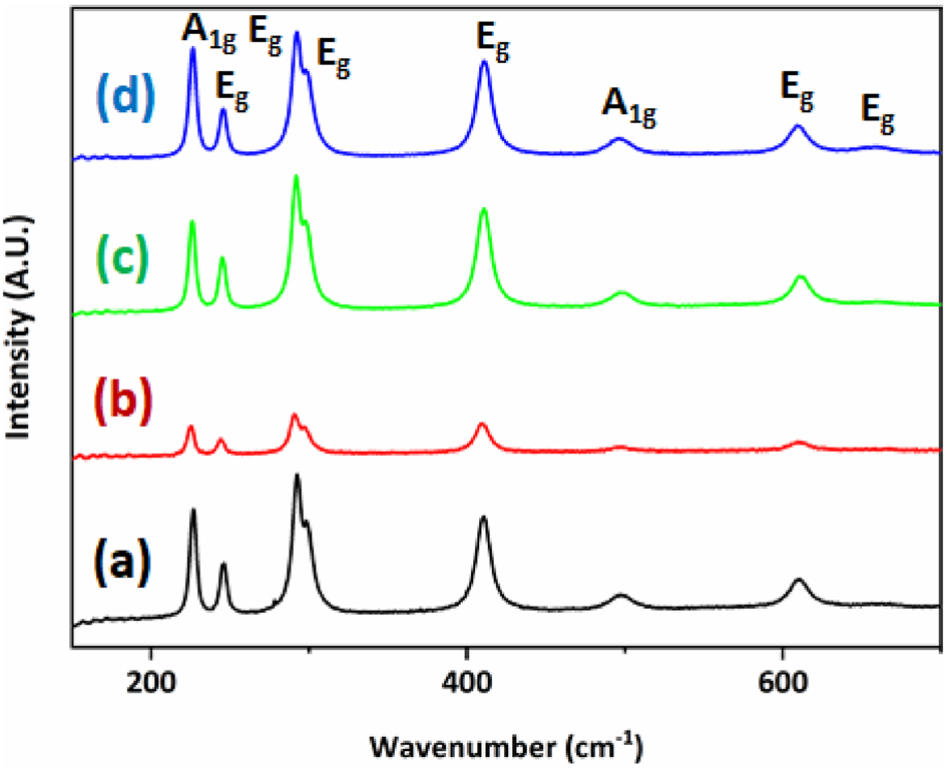
Raman spectra of Cu^2+^ ion doped hematite thin film with pristine (black), 1 mol% (red), 3 mol% (green), and 5 mol% (blue).

The change of micro-crystal structure in Cu^2+^ ion doping into hematite film was confirmed from the Raman spectra as presented in Fig. 5. Hematite has the corundum structure, and it belongs to the D3d point group. Only two A1g modes and five E_g_ modes are Raman active, while two A2u modes and four E_u_ modes are infrared active only^28–31^. For a molecule to be Raman-active, there must be a change in the polarizability of the molecule while IR-active modes are induced by dipole moment change within the molecule. IR active mode could be seen into the Raman spectrum with significantly lower intensity. The polarizability is proportionally changed between the induced dipole moment and the electric field inducing it. The Raman selection rule is defined as δα/δq ≠ 0 where α is the polarizability and q are displacement in the inter-nuclear distance. If the polarizability ellipsoid is changed in size or shape because of molecular vibration, the Raman spectrum will appear. Seven Raman-active bands were found in the range of 200–700 cm^−1^, which is A1g (222 and 496 cm^−1^) and Eg (242, 291, 408 and 610 cm^−1^)^28–30^. However, IR-active Eu mode was found at 660 cm^−1^ at 5 mol% Cu^2+^ ion doped hematite thin film in Fig. 5. This extra peak at 660 cm^−1^ seems to be related with disorder effects due to Cu^2+^ ion doping^20^. Eu mode originates from Fe–O–Fe asymmetric bending. As Cu^2+^ was doped in the crystal lattice of hematite thin film, Fe^3+^ ion was substituted with Cu^2+^ ion, inducing defects in the lattice. Cu^2+^ ion replacement of Fe^3+^ ion into hematite thin film causes δα/δq ≠ 0. In other words, the change of polarizability ellipsoid is giving rise to Eu mode in Raman spectra. It appeared to be a very weak peak at 660 cm^−1^ because the crystal structure distortion of hematite thin film induced by Cu^2+^ ion dopants^28–30^.

**Figure 6:**
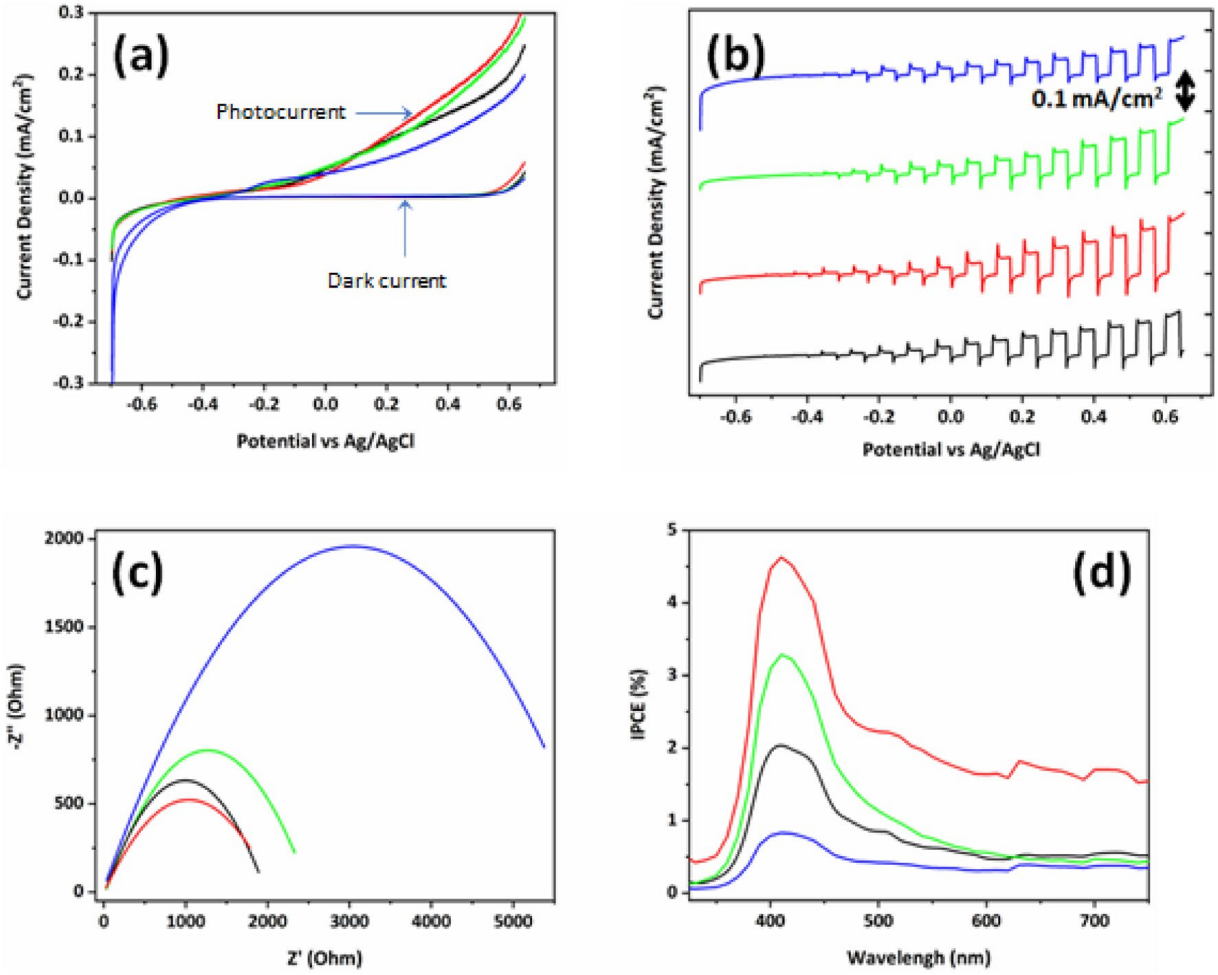
Photoelectrochemical performances of Cu^2+^ ion doped hematite thin film with pristine (black), 1 mol% (red), 3 mol% (green), and 5 mol% (blue). (**a**) LSV measurement, (**b**) chopped photocurrent measurement, (**c**) electrochemical impedance spectroscopy spectra and (**d**) IPCE of Cu^2+^ ion doped hematite thin films.

The photoelectrochemical photocurrent performance with linear and chopped incident light was compared with the prepared four types of hematite thin film in Fig. 6a,b. Pristine hematite showed 0.1 mA/cm^2^ under 1-sun irradiation. 1 mol% of Cu^2+^ ion doped hematite film showed 0.15 mA/cm^2^ at 0.62 V, indicating 50% of increase in photocurrent density. However, 3 mol% and 5 mol% of Cu^2+^ ion doped hematite thin film showed 0.13 mA/cm^2^ and 0.9 mA/cm^2^ at 0.62 V, respectively. It shows that over 1 mol% of Cu^2+^ ion dopants showed slightly decreased current density. Especially, it shows spikes with slightly higher intensity when light is ON and OFF in 1 mol% doped hematite film. This spike is related to electron–hole recombination on the surface of the electrode. This photocurrent value further decreases continuously with increasing Cu^2+^ ion amount to 3 and 5 mol% due to higher charge recombination because of lower electron/hole diffusivity compared to 1 mol% Cu^2+^ ion doped hematite electrode. For better understanding on photocurrent behaviour, electrochemical kinetics by the electrochemical impedance spectroscopy (EIS) was monitored to get Nyquist plots as shown in Fig. 6c. Based on EIS theory on conduction mechanisms of electronic and ionic species at the electrode and electrolyte interface, the obtained EIC semicircles are related to Helmholtz capacitance and charge transfer resistance (R_ct_) on the electrodes surface. It is worthy noted that 1 mol% Cu^2+^ ion doped hematite film show lowest R_ct_ and highest R_ct_ was recorded in 5 mol% Cu^2+^ ion doped film. The pristine hematite thin film and 1 mol% Cu^2+^ ion doped hematite thin film shows almost similar charge transfer resistance (~ 2000 Ωcm^−2^) which is relatively lower than that of 3 mol% (~ 2500 Ωcm^−2^) and 5 mol% (~ 5500 Ωcm^−2^) Cu^2+^ ion doped hematite thin film electrode. Together with photocurrent density and EIS monitoring, the incident photon to current conversion efficiency (IPCE) plots measured at 0.23 V vs Ag/AgCl for all kinds of the prepared electrodes in Fig. 6d. IPCE depends upon the three important efficiencies for photon absorption such as efficiency related to electron–hole separation (η_e-/h+_), efficiency of charge transfer from solid to solid–liquid interface (η_transport_) and efficiency of charge transport through the solid–liquid interface (η_interface_) as shown in equation below^32,33^.

1 $$IPCE = \left( {\eta_{{e^{ - } /h^{ + } }} } \right)*\eta_{transport} *\eta_{interface}$$

It indicates that 1 mol% Cu^2+^ ion doped hematite thin film produced highest IPCE value of 4.7% compared to pristine hematite (~ 2%), 3 mol% (~ 3.3%) and 5 mol% (~ 0.8%) Cu^2+^ ion doped hematite thin film electrodes at 410 nm wavelength.

**Figure 7:**
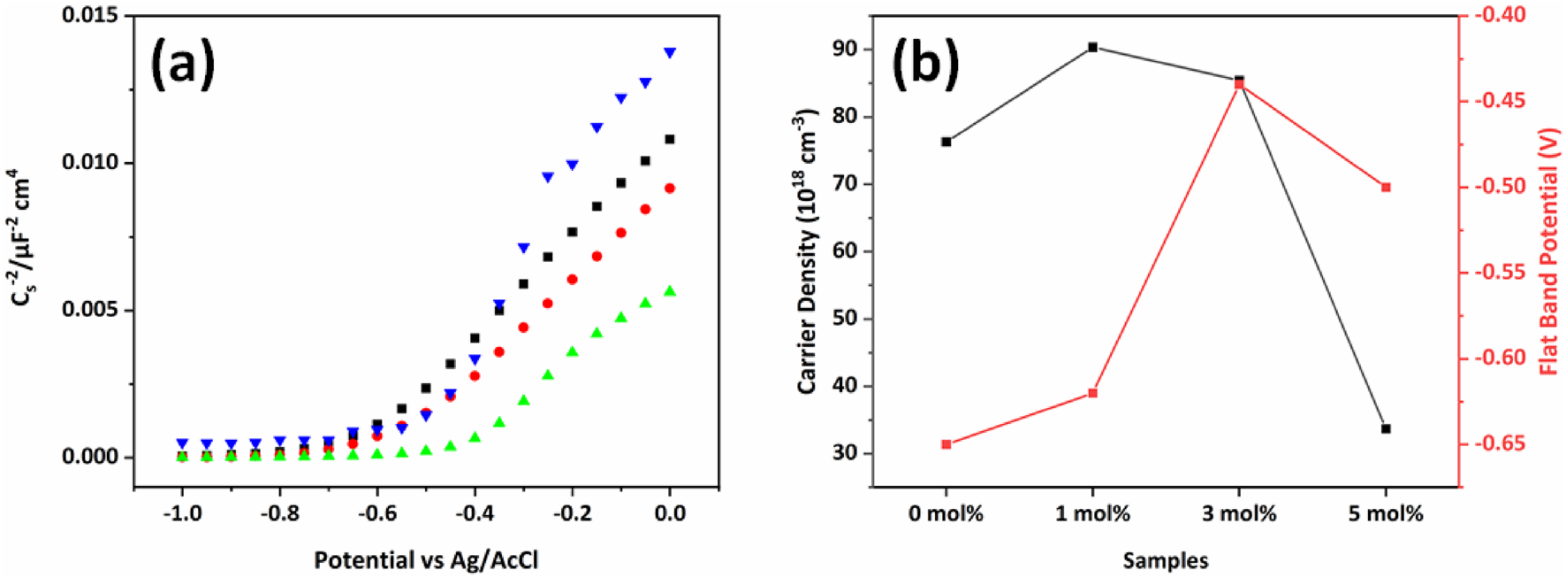
(**a**) Mott-Schottky plots for Cu^2+^ ion doped hematite thin films with pristine (black), 1 mol% (red), 3 mol% (green), and 5 mol% (blue). (**b**) Calculated carrier density and flat band potential value from Mott-Schottky plot.

Even if it is well-known that hematite photocatalyst is a N-type semiconducting material, inherent N-type conductivity is influenced by the dopant. In this point of view, their inherent conductivity depending on dopant was monitored by Mott-Schottky plots in Fig. 7a. From the slope of Mott-Schottky plot, the flat band potential and carrier density of these samples were calculated using the Mott-Schottky equation

2 $$\frac{1}{{C^{2 } }} = \left( {\frac{2}{{e\varepsilon \varepsilon_{0} N_{d} }}} \right)\left( {V_{A} - V_{0} - \frac{{K_{B} T}}{e}} \right)$$

where C is the space charge layer capacitance, e is the electron charge, ɛ is the relative permittivity of hematite (ɛ = 80)^34^, ε_0_ is the permittivity of vacuum, N_d_ is the donor density in cm^−3^, V_0_ is the flat band potential and V_A_ is the applied bias at the electrode. All the samples show a positive plot in the Mott-Schottky graph, as expected from hematite thin film as a n-type semiconductor. However, as the concentration of Cu^2+^ ion dopant increases the plot starts to be reversed and it decreases n-type semiconductor properties and increases p-type semiconductor properties. This observation is consistent with the increase of photocurrent density in negative bias from linear sweep voltammetry in Fig. 6a. The calculated carrier density and flat band potential are compared with amount of dopant in Fig. 7b. The donor density increases for 1 mol% and 3 mol% Cu^2+^ ion doped hematite thin films and decreases for 5 mol% Cu^2+^ ion doped hematite thin films. Therefore, doping with Cu^2+^ ion into hematite thin film could increase the amount of charge carrier density which corresponds to increase in photocurrent density. The high concentration of Cu^2+^ ion doping into hematite thin film decreased PEC activity due to the defects induced by crystal structure distortion of hematite thin film. It suppresses the electron/hole diffusion and enhances the charge recombination in the hematite thin film. This also suppresses the hole transport through the interface of hematite thin film/electrolyte due to the defect at interface, which increases the interface energy barrier. The flat band potential also shifts to a more positive value, indicating that more energy is needed to start electron transfer effectively. This phenomenon was also caused by crystal structure distortion due to defect formation of the hematite unit crystal with Cu^2+^ ion doping.

## Conclusion

Cu^2+^ ion doping into hematite thin film enhances the diffusivity of electron and hole and it also changes the electrochemical properties from n-type to p-type semiconductor. It depends on the formation of crystal defects resulting in the change of surface dipole moment and surface polarizability of hematite thin film. Defects induce the change of dipole moment and stress in crystal lattice of hematite thin film, which was confirmed by Raman spectra, proving the existence of defects from the rise of Raman-inactive E_u_ mode as increasing doping amount of Cu^2+^ ion. In this study, we observed that PEC value was increased for 1 mol% Cu doped hematite film due to shift valence band position upward and increase electron–hole separation rate, which has confirmed by EIS data. The further increment of Cu doping in hematite film shows the decreasing photocurrent values. It was studied by investigating the basic mechanism and process of photon absorption and electron diffusion in the hematite thin film. The increasing amount of Cu^2+^ ion doping into hematite thin film induces crystal structure distortion to produce the defects, decreasing average d-spacing value and IPCE value. It also increases the impedance at the interface of hematite thin film/electrolyte due to enhanced energy barrier by the interface defects. This eventually leads to a reduced photocurrent value. It is concluded that a decreased lattice parameter causes lattice distortion and results in the defects as Cu^2+^ ion replaces Fe^3+^ ion in the crystal lattice of hematite. This produces electron/hole trapping sites and thus diffusivity of electrons and holes is suppressed. This study revealed that he moderate concentration of Cu doped hematite film can be one of the prominent materials for the water splitting application.

## Supplementary Information

Supplementary Information.
