# Supplementary material for: Defectronics based photoelectrochemical properties of Cu2+ ion doped hematite thin film
Source: Sci Rep. 2022 Dec 5;12:20972. doi: 10.1038/s41598-022-20045-6 (PMC9723142; doi:10.1038/s41598-022-20045-6)
Supplement: Supplementary file 1 — Supplementary Information. [file 41598_2022_20045_MOESM1_ESM.docx]

**Electronic Supporting Information**

**Defectronics Based Photoelectrochemical Properties of Cu^2+^ Ion Doped Hematite Thin Film**

*Chang Woo Kim,^†,ǂ^ Amol U. Pawar,**^α,ǂ^ Thomi Hawari, ^§,ǂ^ Na Hyeon Ahn,^§^ Don Keun Lee,^α^ Long Yang,^α^ Ramesh Poonchi Sivasankaran,^α^ Jun Tang,* ^β^ *Zhongbiao Zhuo,* ^β^ *and Young Soo Kang ^α,*^*

*^†.^ Department of Nanotechnology Engineering, College of Engineering, Pukyong National University, Busan, 48513, Republic of Korea.*

*^α.^ Environmental and Climate Technology, Korea Institute of Energy Technology (KENTECH), Naju-Si, Jeollanamdo 58219.*

^§.^ Department of Chemistry, Sogang University, Seoul, 04107, Republic of Korea.

^β.^ Zhejiang Coloray Technology Development Co., Ltd., No. 151, Huishan Road, Deqing County,

Huzhou 313200, China.

^ǂ.^ Authors have an equal contribution.

*Corresponding author

Email: [yskang@kentech.ac.kr](mailto:yskang@kentech.ac.kr)

Telephone: +82-61-3330-9689


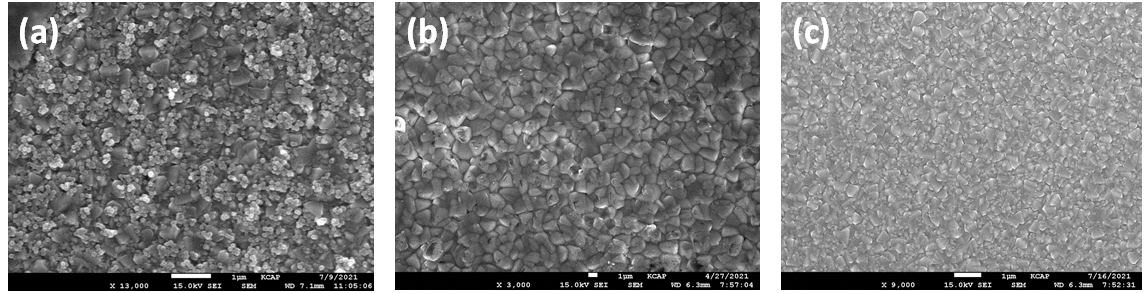


**Figure S1.** Top view SEM images of (a) before and (b) after the secondary growth of pristine hematite thin film, and FTO layer on substrate.


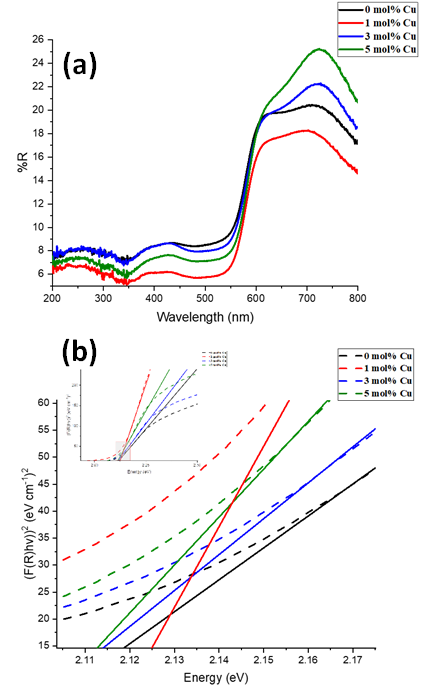


**Figure S2.** (a) UV vis reflectance spectra of 0, 1, 3, and 5 mol% Cu^2+^ ion doped hematite thin films. (b) The band gap energy of 0, 1, 3, and 5 mol% Cu^2+^ ion doped hematite thin film taken from the points where the tangent line intercepting X-axis, indicating 2.118, 2.124, 2.114, and 2.112 eV, respectively, the inset is showing zoomed out version.


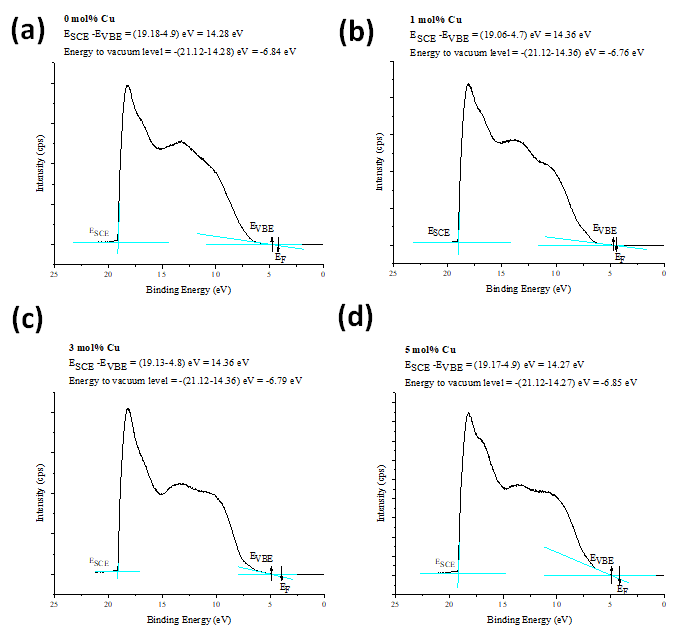


**Figure S3.** UPS spectra of (A) 0 mol%, (b) 1 mol%, (c) 3 mol%, (d) 5 mol% Cu^2+^ ion doped hematite thin films. E_VBE_, E_SCE_ and E_F_ indicate valence band energy, secondary cutoff energy and Fermi level energy, respectively.


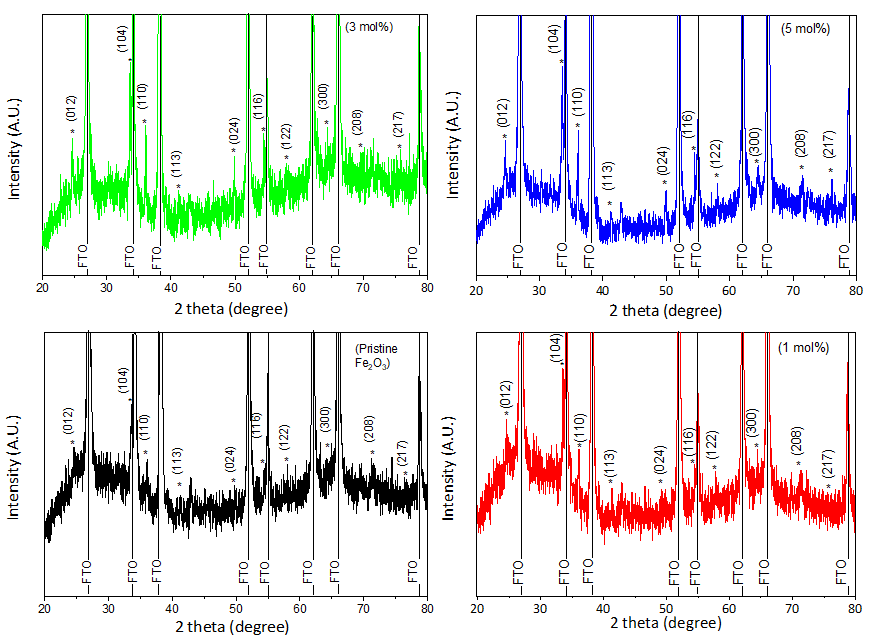


**Figure S4.** The zoom X-ray diffraction patterns of pristine hematite thin film (a, black) and Cu^2+^ ion doped hematite thin film with 1 mol% (b, red), 3 mol% (c, green), and 5 mol% (d, blue) of Cu.

**Table S1.** Crystallite size of pristine hematite and Cu doped hematite films were calculated by using XRD spectra with the help of Debye Scherrer formula. Lattice parameters referred from Hematite standard JCPDS card number 00-001-1053.

| **Sample Name** | **Theta(θ)** | **cosθ** | **β (rad)**  **FWHM** | **Crystallite size (nm)** | **Lattice Parameters**  **a = b, c** | |
| --- | --- | --- | --- | --- | --- | --- |
| **Pristine Fe_2_O_3_** | 16.79 | 0.95737 | 0.005756667 | 25.14852 | a = b=  5.02 Å | C =  13.73 Å |
| **1 mol% Cu doped Fe_2_O_3_** | 16.77 | 0.9574 | 0.003663333 | 39.51494 |  |  |
| **3 mol% Cu doped Fe_2_O_3_** | 16.82 | 0.9572 | 0.004186667 | 34.58468 |  |  |
| **5 mol% Cu doped Fe_2_O_3_** | 16.78 | 0.9574 | 0.005756667 | 25.14719 |  |  |
